# Transcranial photobiomodulation therapy with 808 nm and 1064 nm can affect different behavior parameters in dogs

**DOI:** 10.1007/s10103-026-04918-0

**Published:** 2026-06-19

**Authors:** J. C. Alves, A. Santos, Nieves Pastor Sirvent, P. Jorge, T. Mendonça

**Affiliations:** 1 Divisão de Medicina Veterinária, Guarda Nacional Republicana, Lisbon, Portugal; 2 Faculty of Veterinary Medicine, Universidade Lusófona, Lisbon, Portugal; 3 I-MVET, Faculty of Veterinary Medicine, Lusófona University, Lisbona, Portugal; 4 Centro de Ciência Animal e Veterinária, Universidade Lusófona, Lisbon, Portugal; 5 Department of Animal Medicine, Facultad de Veterinaria, Universidad de Extremadura, Cáceres, Spain

**Keywords:** Photobiomodulation, Canine Frustration Questionnaire, CFQ, Dog Impulsivity Assessment Scale, DIAS, Lincoln Canine Adaptability Resilience Scale, L-CARS, Positive and Negative Activation Scale, PANAS

## Abstract

Objective: To evaluate if transcranial photobiomodulation could improve behaviors in working dogs. Procedures: Thirty-six animals were selected and divided into a no-intervention group (CG, n = 12) and two photobiomodulation groups (TG808 and TG1064, n = 12 for each). The Canine Frustration Questionnaire (CFQ), Dog Impulsivity Assessment Scale (DIAS), Lincoln Canine Adaptability Resilience Scale (L-CARS), and Positive and Negative Activation Scale (PANAS) were the selected outcome measures. They were collected on days 0, 8, 15, 30, 60, and 90 after the initial treatment. At each evaluation time point, group improvements were compared using the Kruskal-Wallis H test, followed by Dunn’s post-hoc test, and the effect size was determined. A significance level of p < 0.05 was set. Results: The animals in the sample had a mean age of 4.4 ± 2.0 years, with 24 males and 12 females. Three breeds were represented: Belgian Malinois Shepherd Dogs (n = 23), Dutch Shepherd Dog (n = 8), and German Shepherd Dogs (n = 5). Changes in the CFQ, L-CARS, and components were mainly driven by improvements in TG1064, with a large effect size. With the DIAS, changes were observed in TG808, with a large effect size. With the PANAS, changes were observed in both groups. Conclusions and Clinical Relevance: This study showed that the described photobiomodulation protocols can improve behaviors in dogs, as evaluated with these behavioral scales. They can have a variety of applications, from behavioral medicine for wellbeing, to the management of hospital or clinical visits.

## Introduction

Dogs are employed in a variety of tasks and missions worldwide. Their roles as service and working animals represent a vital contribution to society and various industries [1]. The path to training a working dog is complex, and several factors can lead to failure in the process. One of the most common reasons for an animal to be dropped from a training program or retired early is behavioral issues, accounting for around 17% of cases [2, 3]. Despite numerous efforts to address this problem persists [4, 5]. While interest in working dogs continues to grow, there remains a lack of detailed information about the specific traits that contribute to a successful working dog. Individual differences in emotional responses lead to variability in performance, suggesting that temperament plays a key role [6].

Temperament can be defined as consistent individual differences in behavioral responses across time and contexts, rooted in affective states and their regulatory processes [7]. Certain aspects of temperament may be particularly important for success in working dogs. Therefore, the management of these animals should include strategies to promote their well-being and overall welfare. The dog’s overall temperament can be influenced by demographic and morphological characteristics, which in turn affect the animal’s ability to cope with various challenges arising from environmental or social interactions [8].

In addition to temperament, specific behavioral traits are also of interest. Frustration is defined as an emotional response that occurs when expectations are violated, or when an animal experiences a reduction or omission of an anticipated reward [9]. It can emerge in situations where the animal is unable to access a desired resource, or when rewards are absent, reduced, or delayed [9]. This emotional state may lead to displacement or repetitive behaviors, and is considered a potential welfare concern [10, 11].

Impulsivity is another key trait, characterized by a reduced ability to tolerate delayed reinforcement. It is closely related to inhibitory control [12] and is associated with a range of behaviors, including aggression, responsiveness to novelty, and general behavioral regulation [12]. Throughout their lives, dogs face various challenges. The ability to recover from adversity or trauma—often referred to as resilience—is crucial [13, 14]. A lack of resilience may contribute to the development of behavioral problems [2].

Several instruments have been developed to assess specific temperament traits in dogs. One such tool is the Positive and Negative Activation Scale (PANAS), adapted from a human version, which evaluates dogs’ responses to rewarding and aversive experiences [8]. This scale is particularly useful for assessing temperament traits [15]. The Canine Frustration Questionnaire (CFQ) is a psychometric tool designed to measure frustration levels in dogs [16]. The Dog Impulsivity Assessment Scale (DIAS) assesses impulsivity in domestic dogs. It has demonstrated good reliability and validity, and its scores correlate with biological markers such as urinary serotonin levels and the serotonin/dopamine ratio [12]. The Lincoln Canine Adaptability Resilience Scale (L-CARS) was developed to evaluate resilience in dogs. It has shown predictive validity for behavioral problems, which are commonly associated with lower resilience [17].

Photobiomodulation therapy (PBMT) is a widely used, non-invasive treatment modality in animal rehabilitation, notable for its absence of adverse effects. It has been effectively applied to manage various conditions in dogs, supported by a robust body of scientific evidence [18–22]. More recently, PBMT has attracted interest for its potential to treat cranial conditions in humans—such as dementia and depression—and in dogs, particularly for addressing cognitive dysfunction [23–26]. The mechanisms behind this effect include stimulation of mitochondrial ATP production in neurons, increased brain blood flow, mitigation of dendritic and neuronal loss, reduced inflammatory and oxidative neuronal damage, and enhanced production of neurotrophic factors [23, 27]. The most commonly used wavelengths for cranial conditions are 808 nm and 1064 nm [28, 29].

We aimed to evaluate whether transcranial PBMT could improve behaviors in working dogs. We hypothesized that PBMT with the two wavelengths can affect different behavior parameters in dogs.

## Materials and methods

In this randomized, double-blinded, placebo-controlled study, the Portuguese versions of the CFQ, the DIAS, the L-CARS, and the PANAS were first translated and validated from the original English versions (not reported here). The original versions of the CFQ (https://ipstore.lincoln.ac.uk/product/canine-frustration-questionnaire-cfq), DIAS (https://ipstore.lincoln.ac.uk/product/the-dog-impulsivity-assessment-scale-dias), L-CARS (https://ipstore.lincoln.ac.uk/product/lincoln-canine-adaptability-scale-l-cars), and PANAS (https://ipstore.lincoln.ac.uk/product/the-positive--negative-activation-scale-panas-for-dogs) are available online. A survey of the working dog population of the Portuguese Gendarmerie was conducted using the Portuguese version of the scales (results not reported here), and a group of target dogs was identified based on results outside the published “normal” range on the different scales. To be included, the dogs must have scores outside the normal range in at least one component in all scales. The animals with most scores outside the normal range were selected.

The animals were active police working dogs, kept in kennels of the Guarda Nacional Republicana, similar in size and construction, located throughout Portugal’s territory. All dogs were fed the same commercially available dog food and, while performing different missions, shared a similar work and exercise background.

The study sample size was determined before the onset of the study through a statistical power analysis (type-1 error, 0.05; type-2 error, 0.8) to determine the minimal number of dogs required to perform statistical comparisons between study groups [30–32]. They were divided into a no-intervention group (CG, *n* = 12) and two PBMT groups (TG808 and TG1064, *n* = 12 for each). CG received no treatment (sham PBMT session with a sham device) on the same days as the treatment for TG808 and TG1064. TG808 and TG1064 received two PBMT sessions per week using a Class IV therapeutic laser (CTS-DUO, Companion Animal Health by Enovis^®^, Lewisville, TX, USA), on non-consecutive days, for 8 consecutive weeks. PBMT parameters are presented in Table 1.

**Table 1:** PBMT parameters

	Light parameters (Dose)	
	TG1	TG2
Wavelength (nm)	808 nm	1064 nm
Radiant Power (W)	3.5	3.5
Irradiance (W/cm2) at skin surface	0.7 (using a large contact treatement head)	0.7 (using a large contact treatement head)
Fluence (J/cm^2^)	5.3	5.3
Total Joules	1050	1050
Treatment Protocol	continuously moving pattern on contact over the entire cranium/calvarium and at the base of the skull and rostral portion of the neck. aiming towards the brainstem/spinal cord at a speed of 2.5–7.5 cm/second. according to manufacturer recommendations	continuously moving pattern on contact over the entire cranium/calvarium and at the base of the skull and rostral portion of the neck. aiming towards the brainstem/spinal cord at a speed of 2.5–7.5 cm/second. according to manufacturer recommendations
Treatment Area (cm^2^)	200	200
Treatment Time	5 min	5 min

Follow-ups were conducted on days 8, 15, 30, 60, and 90 after the initial treatment, using the same scales. A link to the scales was sent via email to the canine handlers, blinded to which group the dogs were randomized into, and the email included a summary of what it involved and an estimated time for completion. All canine handlers who completed the scales were over 18 years old. Informed consent was obtained.

Data were assessed for normality using the Shapiro–Wilk test. At each evaluation time point, group improvements were compared using the Kruskal-Wallis H test, followed by Dunn’s post-hoc test. The effect size was determined with Cohen’s d, and considered to be small if *r* < 0.3, medium if 0.3 < *r* < 0.5, and large if *r* > 0.5. All data were analyzed with IBM SPSS Statistics version 27, and a significance level of *p* < 0.05 was set.

## Results

The animals in the sample had a mean age of 4.4 ± 2.0 years, with 24 males and 12 females. Three breeds were represented: Belgian Malinois Shepherd Dogs (*n* = 23), Dutch Shepherd Dog (*n* = 8), and German Shepherd Dogs (*n* = 5). All animals were followed until the final evaluation. During this period, no changes were introduced in the animals’ normal routines other than the PBMT sessions. There were no significant differences between groups with either questionnaire at the first evaluation point.

Results for the overall score and the five principal components of the CFQ are presented in Table 2. With the CFQ, higher scores, above the normal range, are usually an indication of problems. Significant differences were observed in the overall score at the last two evaluation moments, with TG808 showing better results than CG at + 60 d (*p* = 0.01) and + 90 d (*p* = 0.03), and TG1080 showing better results than CG at + 90 d (*p* = 0.04). With PC1 (general frustration), a difference was observed only at + 60 d, motivated by an increase in score in the CG, difference was observed only at + 60 d, motivated by an increase in score in the CG, which showed a difference to TG808 (*p* = 0.02). An increase was also seen in TG1064 at + 60d. However, this seems to be a transient increase for TG1064, as the score reduced again at + 90d. PC3 (unmet expectations) showed the largest number of differences, from + 8 to + 90 days. These differences were driven by a reduction in TG808 scores, showing better results than CG at +8d (*p* < 0.01), + 15 d (*p* < 0.01), + 30 d (*p* < 0.01), and + 60 d (*p* = 0.04). After discontinuation of treatment (+ 90 d), the differences remained, though lower, for TG808 (*p* = 0.03) and TG1080 (*p* < 0.01). For autonomous control (PC4), changes were observed at +8d, + 30 d, and + 60 d, driven by an early reduction in score in TG1064 at +8d (*p* = 0.01), + 30 d (*p* = 0.03), and + 60 d (*p* = 0.02). Changes were also observed with TG808 at + 30 d (*p* = 0.03) and + 60 d (*p* = 0.02). With PC5 (frustration coping), changes were observed only at the last evaluation point, with a significant reduction in TG808 scores (*p* = 0.02). All differences showed a high effect size.

**Table 2:** Median, inter-quartile range, and percentual change in Canine Frustation Questionnaire (CFQ) scores, by group and moment. Results for overall score and five principal components (PC): “general frustration” (PC1), “barrier frustration/perseverance” (PC2), “unmet expectations” (PC3), “autonomous control” (PC4), and “frustration coping” (PC5), are presented. * indicates significant difference. The normal range is presented in the measure column

Measure	Group	Med T0	IQR	p	ES	Med	+8d IQR	Δ (%)	p	ES
Overall (0,45±0,12)	Control TG808 TG1064	0,66 0,53 0,55	0,26 0,13 0,09	0,09	0,86	0,67 0,52 0,55	0,25 0,04 0,10	2,5-2,2 0,2	0,07	0,81
PC1 (0,38±0,15)	Control TG808 TG1064	0,68 0,460,48	0,32 0,27 0,12	0,09	0,85	0,74 0,48 0,50	0,32 0,06 0,26	8,8 4,3 4,2	0,07	0,85
PC2 (0,55±0,18)	Control TG808 TG1064	0,650,55 0,63	0,33 0,130,18	0,58	0,50	0,650,63 0,65	0,33 0,16 0,26	0,0 13,6 4,0	0,49	0,48
PC3 (0,52±0,17)	Control TG808 TG1064	0,80 0,550,70	0,180,18 0,13	0,06	0,38	0,80 0,58 0,70	0,26 0,18 0,15	0,0 4,50,0	0,03*	0,92
PC4 (0,37±0,13)	Control TG808 TG1064	0,58 0,44 0,44	0,31 0,110,23	0,16	0,82	0,58 0,460,42	0,24 0,08 0,15	0,0 4,5-4,5	0,04*	0,93
PC5 (0,46±0,16)	Control TG808 TG1064	0,60 0,47 0,57	0,35 0,15 0,23	0,16	0,42	0,630,470,67	0,220,15 0,28	5,6 0,0 17,6	0,13	0,45

Med	+15d IQR	Δ (%)	p	ES	Med	+30d IQR	Δ (%)	P	ES	
0,67 0,510,55	0,300,07 0,08	2,5-3,1 0,2	0,16	0,95	0,67 0,51 0,55	0,29 0,06 0,09	2,5-4,0 0,2	0,02*	0,81	
0,780,48 0,50	0,37 0,11 0,24	14,7 4,34,2	0,24	0,39	0,78 0,48 0,54	0,34 0,10 0,13	14,7 4,3 12,5	0,10	0,89	
0,650,63 0,55	0,330,19 0,20	0,0 13,6–12,0	0,34	0,21	0,60 0,60 0,60	0,35 0,06 0,15	−7,7 9,1–4,0	0,31	0,47	
0,80 0,53 0,70	0,32 0,08 0,11	0,0–4,50,0	<0,01*	0,91	0,80 0,55 0,63	0,26 0,20 0,16	0,00,0–10,7	<0,01*	0,81	
0,580,44 0,42	0,36 0,06 0,14	0,00,0–4,5	0,08	0,98	0,60 0,44 0,46	0,26 0,04 0,17	3,4 0,0 4,5	0,14	0,94	
0,670,57 0,60	0,28 0,20 0,20	11,121,4 5,9	0,39	0,23	0,63 0,470,50	0,28 0,17 0,20	5,6 0,0–11,8	0,01*	0,81	

Results for the overall score and the three DIAS factors are presented in Table 3. With the DIAS, higher scores above the normal range are worse. Significant differences were observed in the overall score at + 30 (*p* = 0.03), + 60 (*p* = 0.04), and + 90 days (*p* = 0.02), driven by a reduction in TG808 score. With F1 (behavioral regulation), changes were observed in almost all follow-up moments, even after treatment was discontinued (+ 90 d), again due to a reduction in score in TG808 at + 8 (*p* < 0.01), + 30 (*p* = 0.03), + 60 (*p* = 0.01), and + 90 days (*p* = 0.01), and in TG1080 at + 60 (*p* = 0.04). A difference in “aggression/response to novelty” (F2) was observed at + 30 d, with a decrease in score in both treatment groups (*p* = 0.04 for TG808 and *p* = 0.03 for TG1080). Although no significant differences were found at + 8 and + 15 d, TG1064 showed lower scores at those time points. All differences showed a high effect size.

**Table 3:** Median, inter-quartile range, and percentual change in Dog Impulsivity Assessment Scale (DIAS) scores, by group and moment. Results for overall score and three principal components: “behavioural regulation” (F1), “aggression/response to novelty” (F2), and “responsiveness” (F3), are presented. * indicates significant difference. The normal range is presented in the measure column

Measure		Group	Med T0	IQR	p	ES	Med	+8d IQR	Δ (%)	p	ES	Med	+15d IQR	Δ (%)	p
DIAS	Overall (0,52±0,10)	ControlTG808TG1064	0,58 0,54 0,54	0,100,060,03	0,24	0,27	0,64 0,51 0,55	0,20 0,11 0,05	9,7-6,9 1,8	0,08	0,98	0,59 0,50 0,56	0,19 0,14 0,04	1,0–7,9 2,8	0,17
	F1 (0,47±0,16)	ControlTG808TG1064	0,61 0,57 0,56	0,08 0,09 0,08	0,24	0,40	0,64 0,500,54	0,23 0,110,15	4,9–12,3-3,6	0,02*	0,84	0,63 0,49 0,56	0,20 0,19 0,17	3,3–14,0 0,0	0,08
	F2 (0,37±0,15)	ControlTG808TG1064	0,60 0,54 0,64	0,11 0,13 0,07	0,04	0,92	0,62 0,54 0,50	0,21 0,21 0,21	3,3-0,3–21,9	0,27	0,28	0,62 0,52 0,56	0,26 0,10 0,18	3,3–4,0–12,5	0,39
	F3 (0,70±0,13)	ControlTG808TG1064	0,470,480,48	0,09 0,10 0,16	0,97	0,45	0,58 0,52 0,55	0,16 0,09 0,11	24,7 8,3 13,9	0,94	0,27	0,58 0,540,52	0,22 0,160,08	24,712,5 7,6	0,56

ES	Med	+30d IQR	Δ (%)	p	ES	Med	+60d IQR	Δ (%)	p	ES	Med	+90d IQR	Δ (%)	p	ES
0,88	0,64 0,49 0,59	0,20 0,08 0,06	9,4–8,9 9,2	<0,01*	0,85	0,640,500,56	0,200,070,10	9,4–7,32,0	0,02*	0,99	0,62 0,470,56	0,200,080,08	5,8–13,02,0	<0,01*	0,87
0,93	0,64 0,48 0,60	0,20 0,08 0,13	4,9–15,8 7,1	0,02*	0,93	0,640,470,56	0,200,090,13	4,9–17,00,0	<0,01*	0,97	0,640,430,55	0,200,080,18	4,9–24,6-1,8	<0,01*	0,90
0,20	0,62 0,50 0,60	0,27 0,10 0,18	3,3–7,7-6,3	0,04*	0,84	0,620,520,58	0,240,120,11	3,3–4,0–9,4	0,06	0,92	0,620,520,64	0,260,030,17	3,3–4,00,0	0,21	0,24
0,31	0,58 0,54 0,55	0,23 0,04 0,09	24,7 12,513,9	0,89	0,38	0,580,600,52	0,220,090,12	24,725,08,3	0,36	0,21	0,580,580,56	0,220,120,04	24,720,816,7	0,98	0,41

**Fig. 1:**
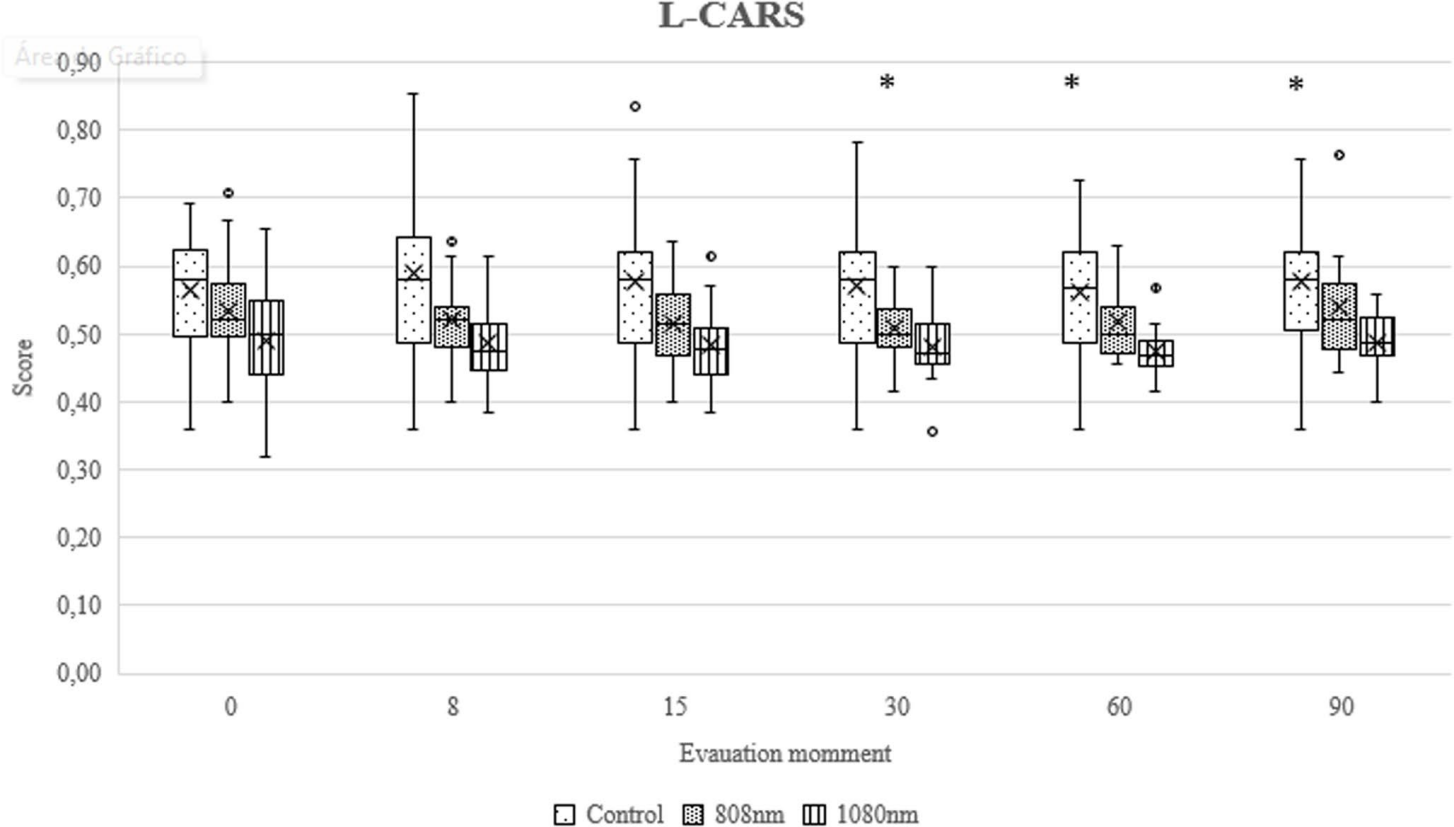
Overall Lincoln Canine Adaptability Resilience Scale (L-CARS), in the no-intervention group (CG) and the two photobiomodulation groups (TG808 and TG1064). Box plots represent median, 25th and 75th percentiles, and whiskers represent 10th and 90th percentiles. * indicates significance

Results for the overall score and the two principal components of the L-CARS are presented in Table 4. Significant differences were observed in the overall score at + 30 (*p* = 0.04), + 60 (*p* = 0.02), and + 90 days (*p* < 0.01), driven by a reduction in TG1080 score, with a high effect size, except at the last evaluation, where a low effect size was observed. With F2, an improvement with TG1080 was observed in + 90 days (*p* = 0.04). The progression of the overall scores are presented in Fig. 1.

**Table 4:** Median, inter-quartile range, and percentual change in Lincoln Canine Adaptability Resilience Scale (L-CARS) scores, by group and moment. Results for overall score and two principal components (PC): “adaptability/behavioral flexibility” (F1), and “perseverance” (F2), are presented. * indicates significant difference. The normal range is presented in the measure column

Measure	Group	Med	T0	IQR	p	ES	Med	+8d IQR	Δ (%)	p	ES	Med	+15d IQR	Δ (%)	p
Overall	Control	0,58		0,13	0,18	0,26	0,58	0,16	0,0	0,07	0,92	0,58	0,14	0,0	0,08
	TG808	0,52		0,08			0,52	0,06	0,0			0,51	0,09	−1,4	
	TG1064	0,50		0,11			0,47	0,07	−5,2			0,48	0,07	−4,5	
F1 (0,82±0,10)	Control	0,59		0,17	0,69	0,45	0,60	0,18	2,4	0,15	0,28	0,60	0,19	2,4	0,22
	TG808	0,55		0,10			0,53	0,04	−3,7			0,53	0,10	−3,7	
	TG1064	0,50		0,15			0,52	0,04	2,2			0,51	0,04	1,1	
F2 (0,81±0,17	Control	0,53		0,17	0,11	0,20	0,53	0,15	0,0	0,13	0,28	0,53	0,15	0,0	0,13
	TG808	0,47		0,17			0,40	0,18	−14,3			0,40	0,22	−14,3	
	TG1064	0,40		0,21			0,37	0,17	−8,3			0,40	0,12	0,0	

ES	Med	+30d IQR	Δ (%)	p	ES	Med	+60d IQR	Δ (%)	p	ES	Med	+90d IQR	Δ (%)	p	ES
0,88	0,58	0,14	0,0	0,03*	0,88	0,57	0,14	−2,1	0,01*	0,86	0,58	0,11	0,0	0,03*	0,9
	0,50	0,05	−4,1			0,50	0,07	−4,2			0,52	0,10	−2,9		
	0,47	0,06	−5,7			0,47	0,04	−6,2			0,49	0,06	2,4		
0,43	0,60	0,19	2,4	0,12	0,82	0,60	0,18	1,5	0,06	0,89	0,60	0,14	−2,1	0,20	0,23
	0,52	0,06	−5,6			0,52	0,06	−4,6			0,54	0,09	2,0		
	0,51	0,06	0,9			0,49	0,08	−2,7			0,51	0,07	0,0		
0,28	0,53	0,13	0,0	0,30	0,49	0,53	0,13	0,0	0,24	0,29	0,53	0,11	0,0	0,02*	0,24
	0,43	0,20	−7,1			0,40	0,15	−14,3			0,47	0,15	0,0		
	0,40	0,18	0,0			0,40	0,22	0,0			0,40	0,10	0,0		

**Fig. 2:**
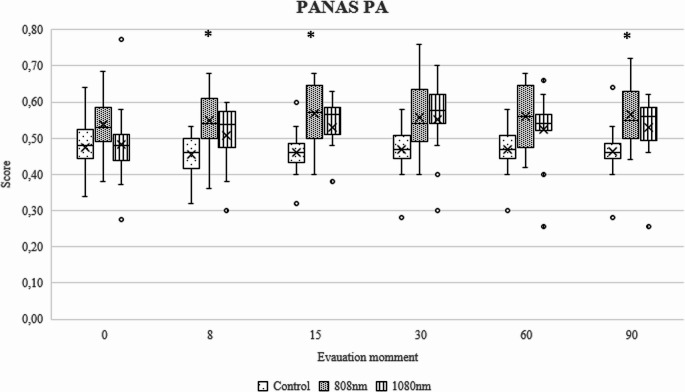
Positive activation (PA) scores of the Positive and Negative Activation Scale (PANAS), in the no-intervention group (CG) and the two photobiomodulation groups (TG808 and TG1064). Box plots represent median, 25th and 75th percentiles, and whiskers represent 10th and 90th percentiles. * indicates significance

Results for the overall PANAS score and its components are presented in Table 5. Significant changes were observed in the overall positive activation score, driven by increases in TG808 scores at +8d (*p* < 0.01), + 15 d (*p* < 0.01), and + 90 d (*p* = 0.02), with a moderate to high effect size. The progression of the positive activation scores are presented in Fig. 2. Changes were also observed in persistence (F2) and excitement (F3). In F2, changes were initially due to a reduction in score in TG808 at + 8 (*p* < 0.01), + 15 (*p* = 0.02), + 30 (*p* < 0.01), + 60 (*p* = 0.04), and + 90 days (*p* = 0.04), and in TG1080 at + 30 d (*p* = 0.03). F3 changed due to variations in the score in TG808 at + 8 (*p* = 0.01), + 15 (*p* = 0.04), + 30 (*p* = 0.04), and + 90 days (*p* < 0.01). In all cases, a high effect size was observed.

**Table 5:** Median, inter-quartile range, and percentual change in Positive and Negative Activation Scale (PANAS) scores, by group and moment. Results for overall negative activation score (NA) and an overall positive activation score (PA), and the three factors of PA: energy and interest (F1), persistence (F2), and excitement (F3), are presented. * indicates significant difference. The normal range is presented in the measure column

Measure	Group	Med	T0	IQR	p	ES	Med	+8d IQR	Δ (%)	p	ES	Med	+15d IQR	Δ (%)	p
NA (0,48±0,15)	Control	0,59		0,13	0,27	0,28	0,53	0,11	−9,7	0,37	0,32	0,58	0,13	−1,5	0,51
	TG808	0,52		0,11			0,57	0,08	9,4			0,61	0,13	16,3	
	TG1064	0,49		0,12			0,57	0,08	15,6			0,59	0,08	19,5	
PA (0,72±0,13)	Control	0,48		0,08	0,25	0,27	0,46	0,09	−4,2	<0,01*	0,40	0,46	0,05	−4,2	0,03*
	TG808	0,53		0,10			0,54	0,11	1,9			0,57	0,14	7,5	
	TG1064	0,48		0,07			0,54	0,10	11,8			0,57	0,21	17,9	
F1 (0,85±0,15)	Control	0,30		0,19	0,64	0,33	0,35	0,11	16,7	0,87	0,32	0,35	0,16	16,7	0,82
	TG808	0,25		0,14			0,38	0,18	50,0			0,38	0,25	50,0	
	TG1064	0,25		0,11			0,38	0,24	50,0			0,40	0,25	60,0	
F2 (0,55±0,18)	Control	0,65		0,16	0,30	0,84	0,60	0,13	−7,7	0,02*	0,98	0,60	0,13	−7,7	0,01*
	TG808	0,75		0,19			0,75	0,09	0,0			0,75	0,16	0,0	
	TG1064	0,70		0,16			0,68	0,10	−3,6			0,65	0,08	−7,1	
F3 (0,79±0,17)	Control	0,35		0,30	0,25	0,29	0,30	0,20	−14,3	0,04*	0,82	0,35	0,15	0,0	0,02*
	TG808	0,45		0,13			0,40	0,10	−11,1			0,50	0,20	11,1	
	TG1064	0,40		0,05			0,40	0,20	0,0			0,40	0,10	0,0	

ES	Med	+30d IQR	Δ (%)	p	ES	Med	+60d IQR	Δ (%)	p	ES	Med	+90d IQR	Δ (%)	p	ES
0,36	0,58	0,07	−1,5	0,78	0,25	0,58	0,10	−1,5	0,39	0,24	0,58	0,12	−1,5	0,38	0,43
	0,56	0,10	7,6			0,58	0,09	11,1			0,59	0,06	12,8		
	0,58	0,11	17,7			0,56	0,11	14,0			0,55	0,09	10,8		
0,83	0,47	0,06	17,7	0,08	0,41	0,47	0,06	−2,1	0,08	0,22	0,46	0,04	−4,2	0,04*	0,94
	0,54	0,15	1,9			0,56	0,17	5,7			0,55	0,13	3,8		
	0,58	0,08	19,9			0,54	0,05	12,5			0,56	0,09	16,7		
0,45	0,35	0,24	16,7	0,62	0,37	0,35	0,21	16,7	0,67	0,26	0,35	0,16	16,7	0,16	0,21
	0,35	0,29	40,0			0,35	0,23	40,0			0,38	0,24	50,0		
	0,45	0,28	80,0			0,50	0,28	100,0			0,48	0,21	90,0		
0,88	0,63	0,13	−3,8	<0,01*	0,88	0,65	0,13	0,0	0,03*	0,98	0,65	0,13	0,0	0,02*	0,82
	0,75	0,13	0,0			0,73	0,16	−3,3			0,73	0,20	−3,3		
	0,70	0,10	0,0			0,70	0,21	0,0			0,65	0,11	−7,1		
0,88	0,30	0,23	−14,3	0,03*	0,82	0,35	0,30	0,0	0,06	0,8	0,30	0,23	−14,3	<0,01*	0,94
	0,50	0,23	11,1			0,50	0,13	11,1			0,50	0,10	11,1		
	0,40	0,15	0,0			0,40	0,13	0,0			0,40	0,10	0,0		

## Discussion

Working and service dogs make a vital contribution to society and different industries. The management of these animals is complex and must include strategies to promote their well-being and overall welfare. Behavior problems are a significant factor in this equation, and, with much still to be discovered in this field, our results indicate that transcranial PBMT can improve behaviors scores in dogs, as measured with various instruments developed to assess specific temperament traits.

Frustration can arise in various situations, including absent, reduced, or delayed rewards, where one is thwarted from achieving a goal [16]. Frustration-related behaviors have been implicated in behavioral problems, including redirected behaviors, repetitive behaviors, aggression, increased arousal, and overall negative affective state [33]. Frustration engages brain regions like the amygdala to trigger stress hormones, while the prefrontal cortex attempts to regulate the response [34]. While the prefrontal cortex is a more superficial area, the amygdala is located more deeply, making it harder to reach. Our results showed that both PBMT protocols improved some frustration-related scores, particularly those related to unmet expectations and frustration coping. In the autonomous control condition, no change was observed in the treatment groups, but the TGs showed some scores above the normal range. This finding is particularly relevant for working dogs, as they are better suited to face challenging conditions and deal with absent, reduced, or delayed rewards. This is also of interest in companion dogs, where frustration can arise in several day-to-day events, and frustration levels are negatively correlated with owner-reported levels of obedience [16].

Impulsivity relates to the ability to make quick, adequate decisions in the face of a changing environment and to an individual’s sensitivity to differences in environmental reinforcement opportunities [1]. Previous reports with successful working dogs have shown that responsiveness is particularly important [1]. Regions implicated in impulsivity include the prefrontal cortex, orbitofrontal cortex, anterior insula, anterior cingulate cortex, striatum, and frontal gyri [35]. This sample of working dogs had mostly normal overall DIAS and factor scores, except “aggression & response to novelty”, where almost all scores were high. Most differences observed with the DIAS were driven by a decrease to normal levels in the animals in TG1064.

Throughout the course of their life, dogs are routinely presented with stressors, such as travel, veterinary visits, hospital stays, and kennelling [17]. Regions implicated in adaptability and resilience include the prefrontal cortex, amygdala, and hippocampus [36]. Curiously, the F1 score for LCARS (adaptability/behavioral flexibility) was below the normal range in all groups and remained unchanged throughout the study. It might be expected that a working dog would score high on this factor, as it is a desired trait. We did observe a change in the overall score, driven by a reduction in score in the two TGs.

Positive emotions primarily activate brain regions like the ventral striatum and medial orbitofrontal cortex, while negative emotions are linked to greater activation in areas such as the amygdala, insula, and dorsomedial prefrontal cortex [37]. The PANAS results were within the normal range at the start of treatment. We observed an increase in positive activation in both treatment groups, starting at the first follow-up and lasting up to + 90 d, even though the treatment had been discontinued by then. We also observed a decrease in excitement (F3) in TG808. While no changes were observed in TG1064, this contrasts with the increase observed in the CG.

The effects of PBMT in brain conditions are numerous, but the stimulation of neuronal mitochondrial ATP production, the increase in blood flow, and reduced inflammation are likely key factors [23]. In fact, there is accumulating evidence to suggest that inflammation is involved in multiple psychiatric disorders, including depression, post-traumatic stress disorder, sleeping disorder, and obsessive–compulsive disorder [38]. This finding is supported by different trials where the administration of omega-3 polyunsaturated fatty acids has beneficial effects on depression, at a comparable level to anti-depressive medication [39, 40]. In animal models, different wavelengths have been used in transcranial PBMT, from around 810 nm and around 1064 nm [41, 42]. In human studies, longer wavelengths, around 1064–1080 nm, have been described [43, 44]. In dogs, a previous report on transcranial PBMT in dogs used a set of different wavelengths ranging from 660 to 905 nm [23]. Wavelength dictates PBMT effects by influencing tissue penetration and specific cellular absorption [45]. Some of the brain areas responsible for the behavioral parameters evaluated in the present study are more superficial, while others are deeper, making them harder to reach. That was one of the reasons for the treatment being the entire cranium/calvarium and the base of the skull, and the rostral portion of the neck, aiming towards the brainstem/spinal cord, in an attempt to cover as large an area and as many structures as possible. Even if a specific area is not reached, they would still benefit from improved blood flow. We observed improvements at both wavelengths, depending on the score considered. In some cases, both groups improved. These findings are likely due to differences in the two wavelengths’ penetration, which can provide guidance on PBMT parameter selection based on clinical findings. Still, as most behaviors are hardly present in isolation, perhaps there is some benefit in combining the two protocols.

The best treatment frequency also has to be determined. Interestingly, we observed early improvements and some degree of persistence in the results observed after the treatments had been discontinued. This is a relevant finding, indicating that patients may enter a maintenance phase after an initial loading phase. Future studies should include a longer follow-up period, both with a maintenance protocol and without treatment. This study has some limitations. The population was composed exclusively of police working dogs. At the same time, it provides a very homogenous sample, with animals of the same breeds, with the same training background, and daily routines. The potential influence of biological variables such as sex should also be evaluated, as the present study was not designed to detect such effects. It would be of interest to compare the results obtained with those in different populations. Also, in contrast to other veterinary questionnaires [46], we do not have a measure of what constitutes a clinically important improvement. Still, the differences observed in this study are reinforced by the large effect size.

## Conclusions

This study showed that the described PBMT protocols can improve behaviors scores in dogs, as evaluated with these behavioral scales. They indicate the potential of a variety of applications, from behavioral medicine for wellbeing, to the management of hospital or clinical visits, which should be explored in future studies.

## Data Availability

All data generated or analyzed during this study are included in this published article.
